# Genetic perspectives on the influence of circulating cytokines on acne: A Mendelian randomization study

**DOI:** 10.1097/MD.0000000000036639

**Published:** 2023-12-15

**Authors:** Jiaxuan Li, Yining Lu, Xuelian Zhao

**Affiliations:** a Department of Plastic Surgery, The Second Hospital of Hebei Medical University, Shijiazhuang, Hebei, P.R. China; b Department of Orthopedic Surgery, The Third Hospital of Hebei Medical University, Shijiazhuang, Hebei, P.R. China.

**Keywords:** acne, biomarkers, GWAS, inflammation, Mendelian randomization

## Abstract

Previous studies have reported that the occurrence and development of acne are closely associated with immune-inflammatory responses. Mendelian randomization was performed to further assess the causal correlation between 41 inflammatory cytokines and acne. Mendelian two-sample randomization utilized genetic variants for acne from a large open genome-wide association study (1299 cases and 211,139 controls of European ancestry) and inflammatory cytokines from a genome-wide association study abstract containing 8293 healthy participants. The causal relationship between exposure and outcome was explored primarily using an inverse variance weighting approach. In addition, multiple sensitivity analyses including MR-Egger, weighted median, simple model, weighted model, and MR-PRESSO were applied simultaneously to enhance the final results. The results suggest that il-10, MIP-1A, and SCGF-β are suggestive of the risk of acne in clinical practice (OR = 0.799, 95% CI = 0.641–0.995, *P* = .045; OR = 0.55, 95% CI = 0.388–0.787, *P* = .001; OR = 1. 152, 95% CI = 1.001–1.325, *P* = .048). Our study conclusively identified a causal relationship between il-10 and circulating levels of acne risk and a suggestive link between MIP-1A and SCGF-β and acne. Our study may provide greater insight into the pathogenesis of acne and develop effective management strategies for the clinic. We believe that IL-10, MIP-1A, and SCGF-β could be potential therapeutic targets for acne development.

## 1. Introduction

Acne is a common skin condition that primarily affects adolescents, especially males. The primary cause of acne is excessive sebum production, which clogs pores and leads to bacterial infection and thickening of the outermost layer of skin, resulting in acne and folliculitis.^[1]^ Additional factors, such as poor lifestyle habits, nutritional imbalances, and fluctuating hormone levels, may also contribute to the development of acne.^[2]^ Although acne is typically not life-threatening, it can significantly impact a person’s quality of life, self-esteem, and psychological well-being. Individuals with acne may experience negative emotions, such as low self-esteem and anxiety, due to changes in physical appearance, which can lead to psychological imbalances or disorders.^[3]^ In addition, in severe cases of scarring acne and folliculitis, acne may become more severe after infection, spreading gradually, and hair or body hair everywhere is also at risk of infection.^[4,5]^

Clinical medicine has recognized acne as a complex and multifaceted disease in which the immune inflammatory response plays a crucial role.^[6–8]^ Studies have shown that Cutibacterium acne interact with the innate immune system through multiple pathways to induce inflammation^[9]^, including through TLR, activation of inflammatory vesicles, induction of matrix metalloproteinase production, and stimulation of antimicrobial peptide activity, in which a fraction of immune cells are also involved.^[10–14]^ Therefore, targeting the altered immune inflammatory response through targeted therapy, including oral medications and topical creams, is crucial in treating acne. It is essential to begin treatment early to reduce the inflammatory response, alleviate pain, and mitigate the impact on the patient’s quality of life.

Mendelian randomization (MR) is a powerful method for causal inference that uses genetic information (especially single nucleotide polymorphisms, or SNPs) to identify instrumental variables for making causal inferences. This approach has become increasingly popular as more genome-wide association analyses have been published.^[15]^ MR is based on 3 assumptions: (1) instrumental variables and exposure factors are closely related, (2) instrumental variables and confounders are not related, and (3) instrumental variables are not directly related to outcomes, and their effects on outcomes can only be expressed through exposure. This method effectively avoids the confounding bias of traditional epidemiological studies.^[16]^

To better understand the causal impact of inflammatory factors on the risk of acne, we applied a two-sample MR approach using pooled statistics on inflammatory cytokines and acne from large cohort genome-wide association studies.

## 2. Method

### 2.1. Data resources

**Figure 1.**
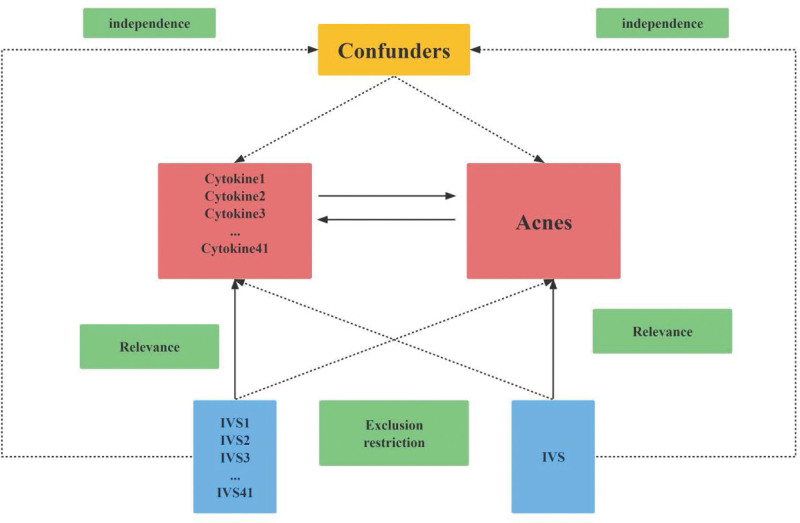
Schematic diagram of the study design in this two-way Mendelian randomization (MR) analysis. Forty-one important instrumental variables for inflammatory cytokines and acne were selected and then explored for bidirectional causality. The 3 basic assumptions of MR analysis, namely correlation, independence, and exclusionary restrictions, are illustrated in this causally directed acyclic graph.

The study design is outlined in Figure 1. Acne cases from a meta-analysis study that included 1299 cases and 211,139 controls of European ancestry. For the genetic instrument of cytokines, summary statistics were taken from the most comprehensive and extensive cytokine genome-wide association study (GWAS); the GWAS cytokine meta-analysis included 8293 Finns from 3 independent population cohorts: the Young Finns Cardiovascular Risk Study, the FINRISK 1997, and the FINRISK 2002 studies^[17]^ (Table 1). All participants provided written informed consent The survey was conducted in Finland, with participants aged 25 to 74 years randomly selected from 5 different geographic regions. Cytokine levels were measured in the participants’ EDTA plasma, heparin plasma and serum. Only measurements within the detectable range of each cytokine were included in the analysis, and any cytokines missing more than 90% of their values (48 of 7) were excluded. We did not require patient consent or ethical approval, the data information was deidentified, and the patient identifiers were removed. Forty-one cytokines were included in this study, including 11 chemokines (CTACK, eotaxin, growth-regulated oncogene-a [GROa], IP10, MCP1, MCP3, monokine induced by interferon gamma [MIG], MIP1a, MIP1b, RANTES, SDF1a), 9 growth factors (bNGF, FGFbasic, GCSF, HGF, MCSF, PDGFb, SCF, SCGFbb, vascular endothelial growth factor [VEGF]), 16 interleukins (IL-10, IL-12p70, IL-13, IL-16, IL-17, IL-18, IL1b, IL1ra, IL-2, IL-2ra, IL-4, IL-5, L-6, IL-7, IL-8, IL-9), and other cytokines (IFNg, MIF, TNFa, TNFb, TRAIL).

**Table 1:** Detailed information of the studies and datasets used in the present study.

Exposures/outcomes	Study/consortium	Participants	Available website
Cytokines	YFS, FINRISK1997, FINRISK2002	8293 Finns	https://www.finngen.fi/fi
Acne	NA	1299 Europeans	https://gwas.mrcieu.ac.uk/datasets/finn-b-L12_ACNE/

### 2.2. Selection of cytokine SNPs

MR analysis has 3 core assumptions, namely correlation, independence, and exclusion restriction.^[18]^ It is assumed that the selected genetic variants are associated with risk factors (correlation) but not with any confounders in the risk factor-outcome association (independence) and that they are not associated with the outcome through any pathway other than the risk factor of interest (exclusion restriction). In this two-way study, 2 GWAS, 41 inflammatory cytokines and acne were utilized. First, we used *P* < 5 × 10^−8^ as a genome-wide significance threshold to select SNPs strongly associated with acne and inflammatory cytokines. second, to avoid linkage disequilibrium, we clustered these SNPs (kb = 10,000, r^2^ = 0.001). palindromic SNPs were discarded because we could not identify these SNPs in exposure and outcome of GWASs in systemic inflammatory regulators were aligned in the same direction. Third, the R^2^ value of each SNP was used to calculate the proportion of variance in exposure, and the F statistic was used to estimate the instrumental strength to avoid weak instrumental bias.^[19,20]^ Finally, we will replace the unavailable SNPs in the result summary with the proxy SNPs (R^2^ > 0.8) from LDlink (LDlink | An Interactive Web Tool for Exploring Linkage Disequilibrium in Population Groups [nih.gov]).^[21]^

### 2.3. Statistical analysis

To evaluate the causal effects of exposures on outcomes, we used a number of complimentary methods in this work, including the inverse variance weighted (IVW), the MR-Egger regression, the weighted median, the simple mode, and the weighted mode methods. The primary analytical method was the IVW approach. When all chosen SNPs were valid IVs, the IVW technique would produce the most accurate findings for basic causal estimations. The Wald ratio estimations are weighted averaged using the IVW technique.^[22]^ Using the premise of Instrument Strength Independent of Direct Effect, the MR-Egger regression runs a weighted linear regression and generates a consistent causal estimate even if all of the genetic IVs are incorrect (InSIDE).^[15]^ It has a poor level of accuracy, though, and is vulnerable to genetic variants. As the weighted median regression technique does not require the InSIDE hypothesis, it is immune to horizontal pleiotropic bias and provides a weighted median of the Wald ratio estimates.^[23]^ It has been established that the Weighted Median technique outperforms the MR-Egger regression in various ways, offering reduced type I error and more causal estimate power. By grouping the SNPs into subsets based on similarities in their causal effects, the causal effect of the subgroup with the most SNPs is calculated using the Weighted Mode approach.^[24]^ The simple mode approach is also less biased than other methods while being less precise since it can reduce bias.^[24]^ Additionally, scatterplots and funnel plots were created for further analysis. The requirement that exposure-related SNPs exclusively affect the outcome through themselves is crucial for the prerequisite of the MR method.

Subsequently, we performed a CochraneQ test on IVW to detect heterogeneity. No heterogeneity was observed for most outcomes, with *P* values >.05. Only a few showed heterogeneity, but our primary MR analysis was IVW; heterogeneity can be present in it, so the presence of heterogeneity in individual outcomes does not have much impact on the prediction of causality.^[25]^

Next, to further assess causality and investigate the presence of pleiotropy, we performed a set of checks, including MR Egger regression and MR-PRESSO.^[26]^In addition, leave-one-out was used to analyze the possibility of individual SNPs confounding the overall MR analysis. We also used PhenoScanner to examine potential dimorphic phenotypes in the evaluated individual SNPs to eliminate their potential influence on the results. All statistical analyses were performed using the “TwoSampleMR” package in R version 3.4.1 (R Foundation for Statistical Computing, Vienna, Austria), and two-tailed *P*-values <.05 were considered statistically significant.

## 3. Result

### 3.1. Causality between IL-10 and acne risks

**Figure 2.**
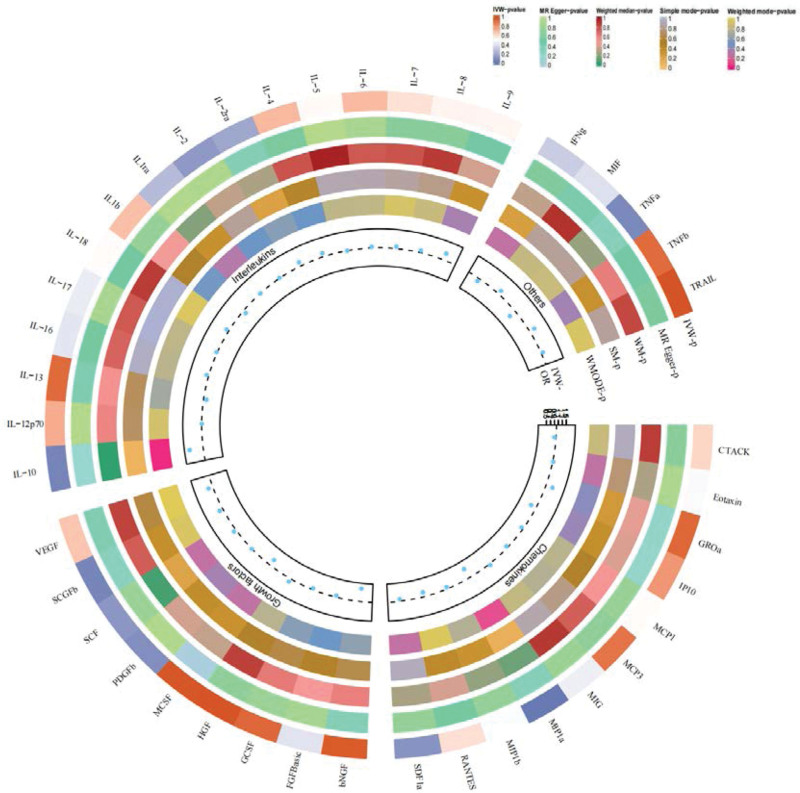
Causal correlations of 41 inflammatory cytokines on acne. The change in the odds ratio (OR) of acnes per one-SD rise in the cytokine level is shown by OR and 95% confidence interval. *P*-value .05/41 = 0.0012 was found significant after multiple-comparison correction. The results from the inverse variance weighted method were shown for all cytokines. bNGF = beta nerve growth factor; CTACK = cutaneous T cell-attracting chemokine; FGFBasic = basic fibroblast growth factor; GCSF = granulocyte colony-stimulating factor; GROa = growth-regulated oncogene-a; HGF = hepatocyte growth factor; IFNg = interferon gamma; IL = interleukin; IP = interferon gamma-induced protein 10; MCP1 = monocyte chemotactic protein 1; MCP3 = monocyte-specific chemokine 3; MCSF = macrophage colony-stimulating factor; MIF = macrophage migration inhibitory factor; MIG = monokine induced by interferon gamma; MIP1a = macrophage inflammatory protein-1a; MIP1b = macrophage inflammatory protein-1b; PDGFbb = platelet-derived growth factor BB; RANTES = regulated upon activation normal T cell expressed and secreted factor; SCF = stem cell factor; SCGFb = stem cell growth factor beta; SDF1a = stromal cell-derived factor-1 alpha; SNPs = single-nucleotide polymorphisms; TNFa = tumor necrosis factor alpha; TNFb = tumor necrosis factor beta; TRAIL = TNF-related apoptosis-inducing ligand; VEGF = vascular endothelial growth factor.*

**Figure 3.**
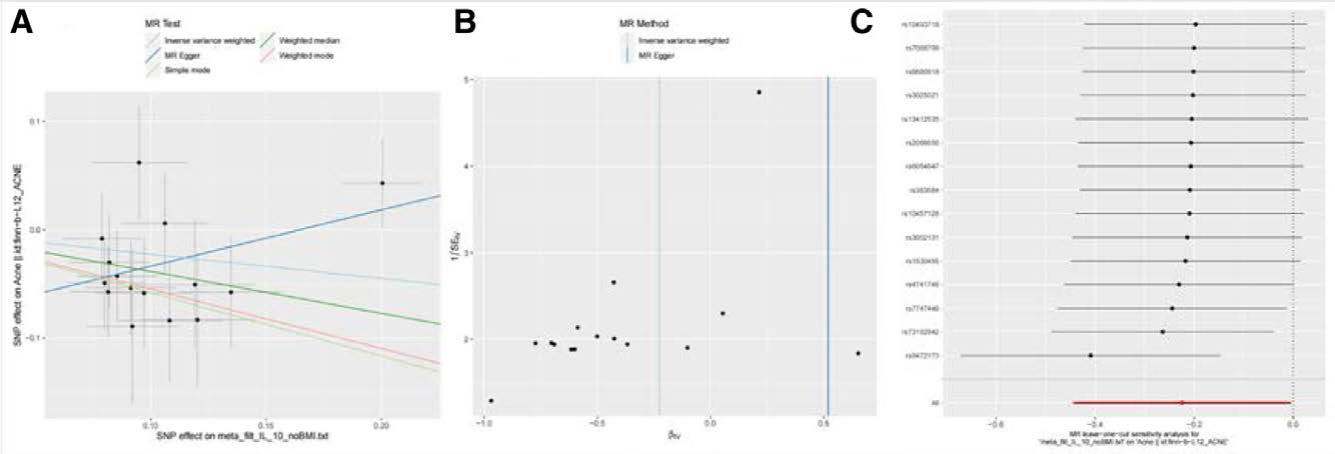
Scatter plots and funnel plots of Mendelian randomization (MR) analyses for IL-10 in acne. (A) Individual inverse variance (IV) associations with cytokine risk are displayed versus individual IV associations with acne in black dots. The 95%CI of the odd ratio for each IV is shown by vertical and horizontal lines. The slope of the lines represents the estimated causal effect of the MR methods. (B) The funnel plots show the inverse variance weighted MR estimate of each cytokine single-nucleotide polymorphism with acne versus 1/standard error (1/SEIV).(C) MR results of leave-one-out sensitivity analysis for acne and IL-10.

A flowchart outlining the full text logic is provided in Figure 1, while the primary MR analysis results for 41 cytokines are presented in Figure 2 and Tables S1–S3, Supplemental Digital Content, http://links.lww.com/MD/L58, http://links.lww.com/MD/L59, http://links.lww.com/MD/L60. Our findings showed that, according to the IVW approach, higher levels of genetic prediction for cyclic 1L-10 were linked to a lower risk of acne (OR = 0.799, 95% CI = 0.641–0.995, *P* = .045 per 1 standard deviation [SD]). We also observed no heterogeneity using Cochran Q test (*P* = .46), and no directional pleiotropy were detected (MR egger-intercept = −0.086, *P* = .051 for MR egger-intercept; *P* = .31 for MR PRESSO global test),the leave-one-out test demonstrated that the MR estimate was stable when individual SNP was removed (Fig. 3, Table S2, Supplemental Digital Content, http://links.lww.com/MD/L59).

### 3.2. Causality between MIP-α and acne risks

**Figure 4.**
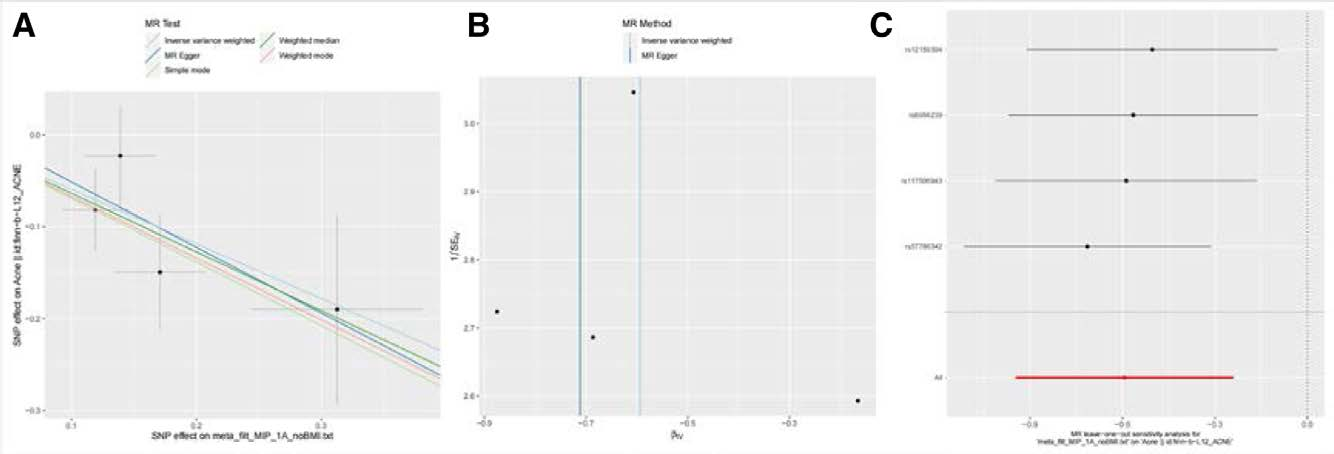
Scatter plots, funnel plots and leave-one-out sensitivity analysis of Mendelian randomization (MR) analyses for MIP-α in acne.

Notably, we also identified a suggestive association between MIP-α in IVW ratio analysis, where we found a suggestive association between circulating levels and reduced acne risk for every 1 increase in SD (OR = 0.55, 95% CI = 0.388–0.787, *P* = .001), with no heterogeneity found by Cochran Q test (*P* = .597). We also did not find any directional pleiotropy, the leave-one-out test demonstrated that the MR estimate was stable when individual SNP was removed (MR egger-intercept = 0.019, *P* = .845 for MR egger-intercept; *P* = .66 for MR PRESSO global test) (Fig. 4, Table S2, Supplemental Digital Content, http://links.lww.com/MD/L59).

### 3.3. Causality between SCGF-β and acne risks

**Figure 5.**
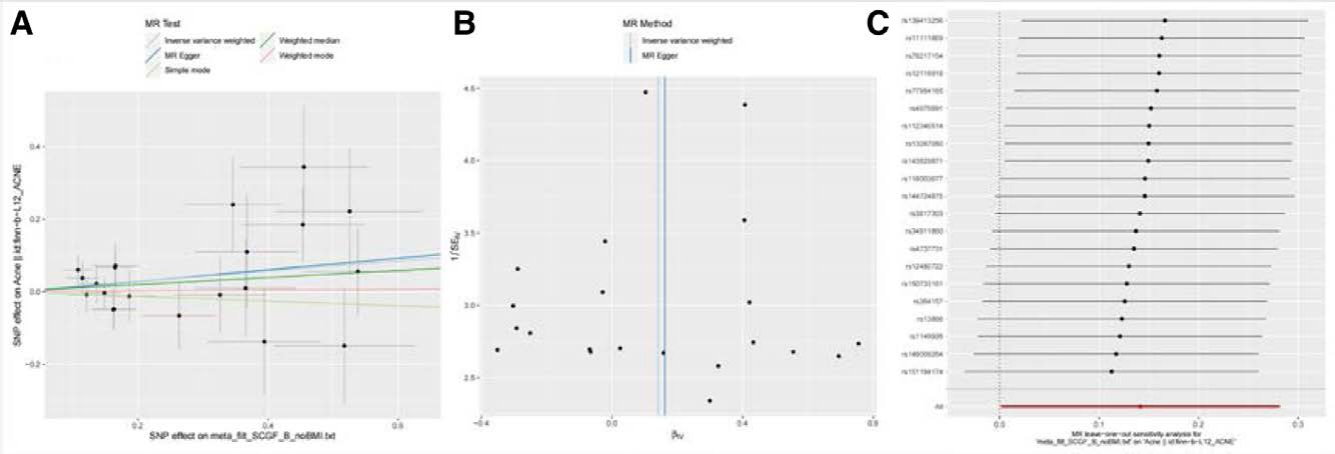
Scatter plots, funnel plots and leave-one-out sensitivity analysis of Mendelian randomization (MR) analyses for SCGF-β in acne.

Moreover, using IVW analysis, we found that increased levels of SCGF-β in circulation were associated with a higher risk of acne (OR = 1. 152, 95% CI = 1.001–1.325, *P* = .048, per 1 standard deviation increase in SCGF), and there was no heterogeneity (Cochrane Q test, *P* = .343). Furthermore, we did not observe any directional pleiotropy (MR egger-intercept = −0.004, *P* = .878 for MR egger-intercept) (Fig. 5, Table S2, Supplemental Digital Content, http://links.lww.com/MD/L59). The leave-one-out test demonstrated that the MR estimate was stable when individual SNP was removed.

Except for IL-10, SCGF-β, and MIF-α, other cytokines (e.g., VEGF, GRO-α, Trail, MIG, IL-7, IL-17) did not exhibit any association with acne risk in the IVW primary MR analysis or other secondary analyses. In our heterogeneity test, we found significant heterogeneity in EOTAXIN (*P* = .003), GROa (*P* = .006), MIG (*P* = .046), RANTES (*P* = .044), while most other cytokines demonstrated significant non-heterogeneity. Our MR-egger regression did not highlight any pleiotropy in P-values for all cytokines. Finally, our MR-PRESSO assay as an additional robustness test did not identify any outliers, except for EOTAXIN (*P* = .01) and GROa (*P* < .01).

## 4. Discussion

Acne is a common skin condition that is caused by a combination of factors including genetics, hormonal changes, certain medications, stress, and dietary factors.^[1,2,27]^ When hair follicles become clogged, they provide an environment for Propionibacterium acnes to grow, triggering an inflammatory response that causes redness and pain, ultimately resulting in the formation of papules.^[1,28]^ Although acne is not life-threatening, it can significantly impact a person’s quality of life, self-esteem, and psychological well-being.^[3,27]^ However, little is known about the biological mechanisms underlying the cause of acne. To address this, we performed a two-sample MR analysis, which is the first MR analysis to determine whether an elevation in inflammatory cytokine levels is associated with an increased risk of acne based on genetic data from publicly available databases. Our findings suggest a causal relationship between high levels of MIP-1A/IL-10 and a reduced risk of acne. Elevated circulating levels of SCGF-β were also suggestively associated with an increased risk of acne.

IL-10 is a 19 kDa lymphokine produced by various cell types such as B and T cells and monocytes. Our study found that high levels of circulating IL-10 were associated with a reduced risk of acne (OR = 0.799, 95% CI = 0.641–0.995, *P* = .045 per 1 SD increase). In normal human skin, this cytokine is not expressed in keratinocytes, but it can be induced after UV-B irradiation^[29]^, IL-10 exerts immunosuppressive and anti-inflammatory effects by inhibiting c-interferon production.^[30,31]^ Acne can affect IL-10 cytokine expression, which has been associated with a good prognosis in acne patients, ^[32,33]^ IL-10 can inhibit the production of c-interferon by producing inflammatory mediators to reduce the inflammatory response, ^[34]^ inhibit apoptosis, and prevent hyperkeratosis.^[35]^ Therefore, IL-10 may reduce the risk of acne through various mechanisms. Kang et al also found that elevated IL-10 in acne may act as an inhibitory mechanism of acne inflammation, further highlighting the potential of IL-10 as a target for acne control.^[36]^

MIP-1α/CCL3 is a member of the chemokine family involved in various biological processes, such as inflammatory responses and immune regulation.^[37]^ Clinical observational studies or meta-analyses linking MIP-1α with acne are limited, but our MR analysis found that high levels of circulating MIP-1α were associated with a reduced risk of acne (OR = 0.55, 95% CI = 0.388–0.787, *P* = .001 per 1 SD increase). Normally, CCL3 plays a pro-inflammatory role, which is detrimental to a good disease prognosis^[38,39]^. However, a recent study showed that in a state of skin inflammation, keratinocytes overexpress CCL3 and recruit T cells to the epidermal region.^[40]^ This interaction can control the recruitment of other chemokines and associated circulating cytokines to mediate the immune response, promote barrier repair, and epidermal duplication, suggesting that the interaction between CCL3 and T cells plays a crucial role in skin barrier repair and normal epidermal cell function in the inflammatory state of the skin.^[41–44]^ Further in-depth studies are needed to understand the activity of the skin immune system comprehensively, including the interaction between CCL3 and T cells in skin inflammatory states, such as acne, to provide new ideas and approaches for the treatment of skin inflammation-related diseases.

SCGF is a cytokine that targets primitive hematopoietic progenitor cells. In our study, high levels of circulating SCGF-β were associated with an elevated risk of acne (OR = 1. 152, 95% CI = 1.001–1.325, *P* = .048, 1 SD per increase in SCGF) and did not show heterogeneity (Cochrane Q test, *P* = .343). There are few studies correlating SCGF-β with skin inflammatory responses. Lan et al suggested that serum SCGF may interact with the stem cell factor pathway on keratinocytes to promote disease progression.^[45]^ Keratinocytes recruit mast cells by secreting stem cell factor, and as a result, mast cells releases inflammatory mediators, including histamine, VEGF, IL-6, and IL-8, which increase endothelial permeability and vasodilation and recruit inflammatory cells to promote inflammatory responses.^[46]^ Currently, we know little about SCGF-β and its role in acne pathogenesis. Our study highlights the potential of SCGF-β as playing an important role in acne pathogenesis. Further studies are needed to reveal its specific mechanism of action and provide new ideas and approaches for the treatment of acne.

Our study has several strengths. (1) This is the first MR study to elucidate the relationship between inflammatory cytokines and risk of acne vulgaris. (2) Unlike observational studies, our current study avoids confounding factors and reverse causality as much as possible, providing a reliable pair of cause-and-effect relationships. (3) Our study data were obtained from the publicly available GWASS database with a large amount of original study data, which provided a strong guarantee for this study. (4) Unlike time-consuming randomized controlled studies (RCTs), the cost of time and money invested in this study is highly cost-effective for the results we obtained. However, this study also has some limitations. First, because the data in the database were from Europe, the study was limited to European participants and the usefulness of the results for other populations remains to be seen. Second, this study ignored the diversity of acne disease and did not analyze multiple subtypes of acne, and cytokines may have a causal effect on acne caused by different etiologies. Third, cytokines are a dynamic indicator, unlike other indicators such as body weight, and MR does not address the dynamics of cytokine levels.

## 5. Conclusion

Our study conclusively identified a causal relationship between IL-10 and circulating levels of acne risk, as well as a suggestive link between MIP-1A and SCGF-β and acne. Our study may provide greater insight into the pathogenesis of acne and develop effective management strategies for the clinic. We believe that IL-10, MIP-1A and SCGF-β could be potential therapeutic targets for acne development.

## Acknowledgments

We are grateful to all those who took part in or assisted with this study project.

## Author contributions

**Writing – original draft:** Li Jiaxuan, Yining Lu.

**Writing – review & editing:** Xuelian Zhao.

## Supplementary Material






